# Co‐Stabilization of High‐Entropy Oxides by Entropy and Polyanionic Units toward High‐Capacity Zero‐Strain Anodes for Advanced Lithium‐Ion Batteries

**DOI:** 10.1002/advs.202516795

**Published:** 2025-11-08

**Authors:** Xiehang Chen, Yang Xiang, Cong Li, Shipai Song, Xincong Liu, Bing Luo, Yong Xiang

**Affiliations:** ^1^ Frontier Center of Energy Distribution and Integration Tianfu Jiangxi Lab Huoju Avenue, Futian Sub‐District, Jianyang city Chengdu Sichuan 641419 P. R. China; ^2^ State Key Laboratory of Electronic Thin Films & Integrated Devices School of Materials and Energy University of Electronic Science and Technology of China 2006 Xiyuan Avenue, West High‐Tech Zone Chengdu Sichuan 611731 P. R. China; ^3^ School of Architecture and Civil Engineering Chengdu University Chengdu Sichuan 610106 P. R. China

**Keywords:** anode materials, high‐entropy oxides, lithium‐ion batteries, oxygen defects, zero‐strain

## Abstract

High‐entropy oxides (HEOs) have attracted considerable attention as anode materials for lithium‐ion batteries, owing to their entropy‐driven structural stabilization. However, practical deployment is often limited by rapid capacity fading and irreversible phase transitions during cycling. To overcome these challenges, a phosphorus doping strategy is introduced that incorporates stable PO_4_^3−^ groups into the HEO lattice, resulting in a novel zero‐strain anode material, denoted as [P_x_(LiCrMnFeCoZn)_1‐x_]_3_O_4_ (PHEO). The PHEO anode delivers a high specific capacity of 686.2 mAh g^−1^ at 0.5 A g^−1^, along with exceptional cycling stability exhibits exceptional cycling stability, retaining 116.4% of its capacity after 200 cycles and 149.6% after 1000 cycles at 2 A·g^−1^. Structural and electrochemical analyses reveal that phosphorus doping effectively modulates the chemical states of multiple cations and enriches oxygen vacancy concentration, which reduces the bandgap and promotes pseudocapacitive charge storage. These effects, combined with the robust PO_4_ tetrahedral framework and high‐entropy effect, collectively contribute to enhanced specific capacity, improved structural integrity during cycling, and facilitated Li^+^ transport kinetics. This work offers a viable strategy for designing high‐capacity, long‐life HEO anodes with zero‐strain characteristics for next‐generation lithium‐ion batteries.

## Graphical abstract

Phosphorus‐doped high‐entropy oxide anode material utilizes entropy stabilization and PO_4_^3−^ polyanionic groups to achieve zero‐strain and exceptional cycling stability. It delivers a high capacity of 686 mAh g^−1^ and retains 149.6% capacity after 1000 cycles at 2 A g^−1^, supported by enhanced charge compensation, oxygen vacancies, and a robust framework for superior Li^+^ transport.

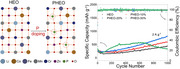

## 1 Introduction

The pursuit of high‐capacity anode materials with minimal volume strain is crucial for next‐generation lithium‐ion batteries (LIBs), particularly for all‐solid‐state configurations where mechanical stability is paramount.^[^ ^1^, ^2^ ^]^ This challenge is illustrated by two dominant categories of anode materials: one offers high specific capacity but undergoes substantial volume variations during cycling, while the other demonstrates exceptional structural stability with near‐zero‐strain yet provides limited capacity. For instance, many conversion‐ and alloying‐type anode materials (e.g., Si, Sn, and transition metal oxides) possess high theoretical capacities but suffer from significant volume expansion, resulting in rapid capacity degradation.^[^ ^3^, ^4^, ^5^ ^]^ In contrast, zero‐strain materials such as spinel Li_4_Ti_5_O_12_, LiCrTiO_4_ and LiY(MoO_4_)_2_ demonstrate outstanding cyclability with minimal unit cell change (< 1%), yet their low capacities and relatively high operating voltages limit their practical application in high‐energy‐density systems.^[^ ^2^, ^6^, ^7^ ^]^ The inherent trade‐off between capacity and mechanical stability remains a major obstacle in the design of advanced battery materials.

High‐entropy oxides (HEOs) have recently emerged as a promising platform for reconciling these competing characteristics. Since the pioneering work by Rost et al. in 2015,^[^ ^8^ ^]^ HEOs have garnered increasing attention in the LIBs community.^[^ ^9^, ^10^, ^11^, ^12^, ^13^ ^]^ By incorporating multiple principal cations into a single‐crystal structure, HEOs achieve high configurational entropy (*S_config_* ≥ 1.5R), which facilitates entropic stabilization, buffers volume variation during lithiation/delithiation, and improves mechanical durability.^[^ ^9^, ^10^, ^14^ ^]^ For instance, Sarkar et al. developed a (Co_0.2_Cu_0.2_Mg_0.2_Ni_0.2_Zn_0.2_)O anode, which exhibited improved cycling stability and capacity retention compared to medium‐entropy analogs, attributed to its higher entropy (1.61R) and the inclusion of electrochemically inert Mg, forming buffering phases.^[^ ^11^, ^15^, ^16^ ^]^ Qiu et al. further demonstrated that the in situ formation of MgO in (CoCuMgNiZn)O effectively mitigates volume changes and nanoparticle aggregation, leading to enhanced cycling and rate performance.^[^ ^17^ ^]^ Moreover, multiple cations in HEOs enable high specific capacity through multi‐electron redox reactions and abundant oxygen vacancies (O_V_), which improve charge transfer and Li^+^ diffusion kinetics.^[^ ^14^, ^18^, ^19^ ^]^ The compositional flexibility of HEOs allows for the strategic selection of transition metals to increase capacity and the use of aliovalent doping to optimize charge compensation, O_V_ formation, and structural stability.^[^ ^20^ ^]^ Such vacancies not only provide additional sites for Li^+^ storage but also promote surface‐mediated pseudocapacitance, significantly improving rate capability and reversible capacity.^[^ ^21^ ^]^ Nevertheless, despite these advantages, many HEO anodes still experience non‐negligible volume changes and capacity decay over long‐term cycling, indicating that entropy stabilization alone is inadequate to achieve both high capacity and near‐zero‐strain.

Non‐metal doping represents a facile yet effective strategy for modulating the physicochemical properties of anode materials and enhancing their performance in energy storage applications.^[^ ^22^, ^23^, ^24^ ^]^ To address the aforementioned limitations, we propose the incorporation of phosphorus (P) into HEOs to leverage the unique properties of PO_4_^3−^ polyanionic units, synergistically improving the capacity and cycling stability. Elemental P, as a non‐metal with high electronegativity and metalloid characteristics,^[^ ^22^, ^25^ ^]^ can promote electron transfer and creates localized regions of electron deficiency and enrichment, thereby enhancing electrochemical activity during Li^+^ insertion/extraction.^[^ ^26^ ^]^ More importantly, PO_4_^3−^ groups form highly robust tetrahedral frameworks that significantly enhance structural integrity, a concept well‐established in polyanion‐type cathode materials such as LiFePO_4_. By combining entropy stabilization with the robustness of polyanion chemistry, P doping offers a promising route to decouple high capacity from large volume strain. In this study, we designed and synthesized a P‐doped high‐entropy oxide anode material, [P_x_(LiCrMnFeCoZn)_1‐x_]_3_O_4_, with the aim of achieving high capacity and zero‐strain. The material exploits the synergistic effects of P doping, including the formation of a stable PO_4_^3−^ tetrahedral framework and the promotion of O_V_, to enhance structural stability and capacity. We systematically investigated the influence of doping content on O_V_ formation, pseudocapacitive behavior, and electrochemical performance. Through ex situ X‐ray diffraction (XRD) and transmission electron microscopy (TEM), we also elucidated the microstructural evolution of the electrode during cycling. This work provides both theoretical and experimental foundations for the design of high‐capacity and zero‐strain anode materials for LIBs and proposes a generalizable strategy for optimizing multi‐element doping in high‐entropy systems.

## 2 Results and Discussion

### 2.1 Structural, Compositional, and Physical Property Analysis

**Figure 1:**
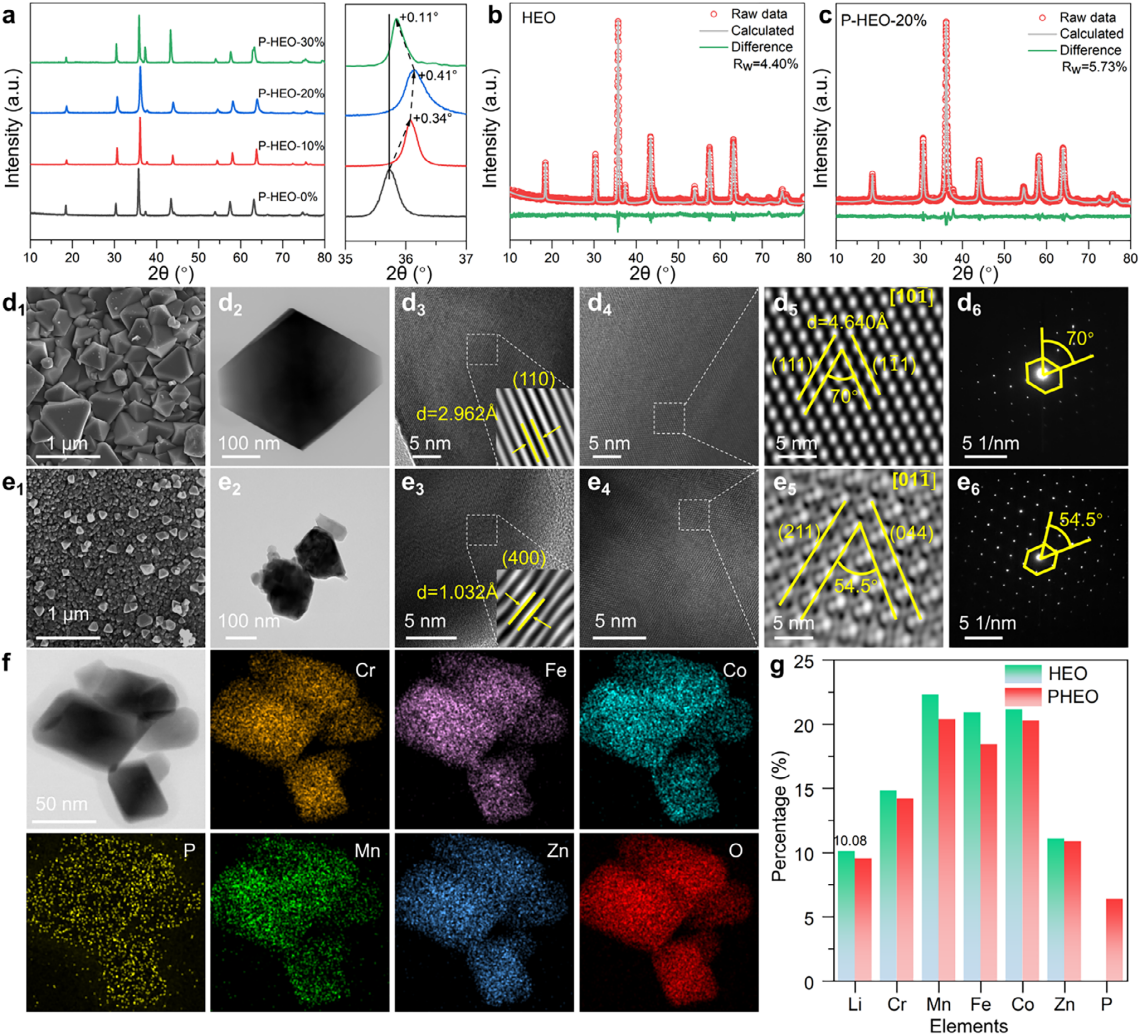
Structural, morphological, and compositional characterization of pristine HEO and PHEOs. a) Wide‐angle XRD patterns demonstrating phase purity, with an inset highlighting the characteristic (311) peak shift. Rietveld refinement results of the XRD patterns for b) pristine HEO and c) PHEO‐20%, revealing detailed crystal structure parameters. d_1_–d_6_) Multiscale morphological and structural analysis of pristine HEO: (d_1_) Representative SEM image, (d_2_) TEM overview, (d_3_,d_4_) High‐resolution TEM images, (d_5_) Locally IFFT filtered pattern indicating lattice fringes, and (d_6_) corresponding SAED pattern. Analogous multiscale characterization for PHEO‐20%: e_1_) SEM, e_2_) TEM, e_3_,e_4_) HRTEM images, e_5_) Local IFFT pattern, and e_6_) SAED pattern. f) HAADF‐STEM image and associated EDS elemental mapping of PHEO‐20%, confirming homogeneous elemental distribution. g) ICP‐OES results quantifying the elemental compositions of both HEO and PHEO‐20%.

HEO and PHEO were synthesized using a sol–gel method followed by calcination (details are provided in Supporting Information; compositions are listed in Table S1, Supporting Information). XRD patterns (**Figure** **1**a) confirm that all materials except PHEO‐30% adopt a pure spinel phase (JCPDS No. 80–1668) with no detectable impurities. Additional diffraction features not associated with the spinel structure are visible in the enlarged patterns (Figure S1, Supporting Information). High‐resolution analysis of the (311) peak reveals a clear shift toward higher angles in PHEO (PHEO‐20%) compared to HEO (PHEO‐0%), indicating lattice contraction due to successful P doping.^[^ ^11^ ^]^ The magnitude of this shift initially increases and then decreases with higher doping levels, as P atoms first occupy larger transition‐metal sites, causing contraction due to their smaller atomic radius. While the doping level reaches 30%, they increasingly occupy smaller Li^+^ sites, resulting in a contraction‐expansion sequence of the lattice.^[^ ^27^ ^]^ Rietveld refinements (Figure 1b,c; parameters are summarized in Table S2, Supporting Information) yield lattice constants of a = b = c = 8.333255 Å, V = 578.69 Å^3^ for HEO, and a = b = c = 8.247279 Å, V = 560.96 Å^3^ for PHEO. Broader diffraction peaks in PHEO further suggest P doping induced lattice distortion and heterogeneous strain. Refinement results for other doping levels are provided in Figure S2 and Table S2 (Supporting Information).

Microstructural analysis of HEO and PHEO (Figure 1d,e) reveals nanoscale particles with distinct size distributions: 100–1000 nm for HEO versus 50–200 nm for PHEO (Figure 1d_1_,d_2_,e_1_,e_2_), confirming that P doping effectively refines particle size. This is a key factor for improving electrochemical performance. SEM‐EDS mapping (Figures S3 and S4, Supporting Information) confirms uniform distribution of transition metals (Cr, Mn, Fe, Co, Zn) in both materials and homogeneous dispersion of P in PHEO. High‐resolution TEM images of HEO (Figure 1d_3_–d_5_) show clear lattice fringes with a spacing of 2.962 Å corresponding to the (110) plane, along with ordered atomic arrangements verified by fast Fourier transform (FFT) and inverse FFT (IFFT). Interplanar spacings and angles are consistent with the spinel structure, as further supported by selected‐area electron diffraction (SAED) patterns (Figure 1d_6_). PHEO (Figure 1e_3_–e_6_) maintains spinel symmetry, exhibiting lattice spacing of 1.032 Å (400), 3.365 Å (211), and 1.457 Å (044) with theoretical interplanar angles, alongside localized lattice distortions induced by P doping. High‐resolution elemental mapping (Figure 1f; Figure S5, Supporting Information) confirms uniform co‐distribution of Cr, Mn, Fe, Co, Zn, P, and O within PHEO crystallites. ICP‐OES results (Figure 1g) indicate that all metallic elements in both materials, as well as P in PHEO, fall within the range of 5–35 at.%, satisfying the high‐entropy oxide criterion and supporting the role of entropy stabilization. These results collectively confirm the successful synthesis of phase‐pure, compositionally uniform P‐doped HEOs, in which P doping reduces particle size and introduces controlled lattice distortions without compromising the integrity of the spinel structure.

**Figure 2:**
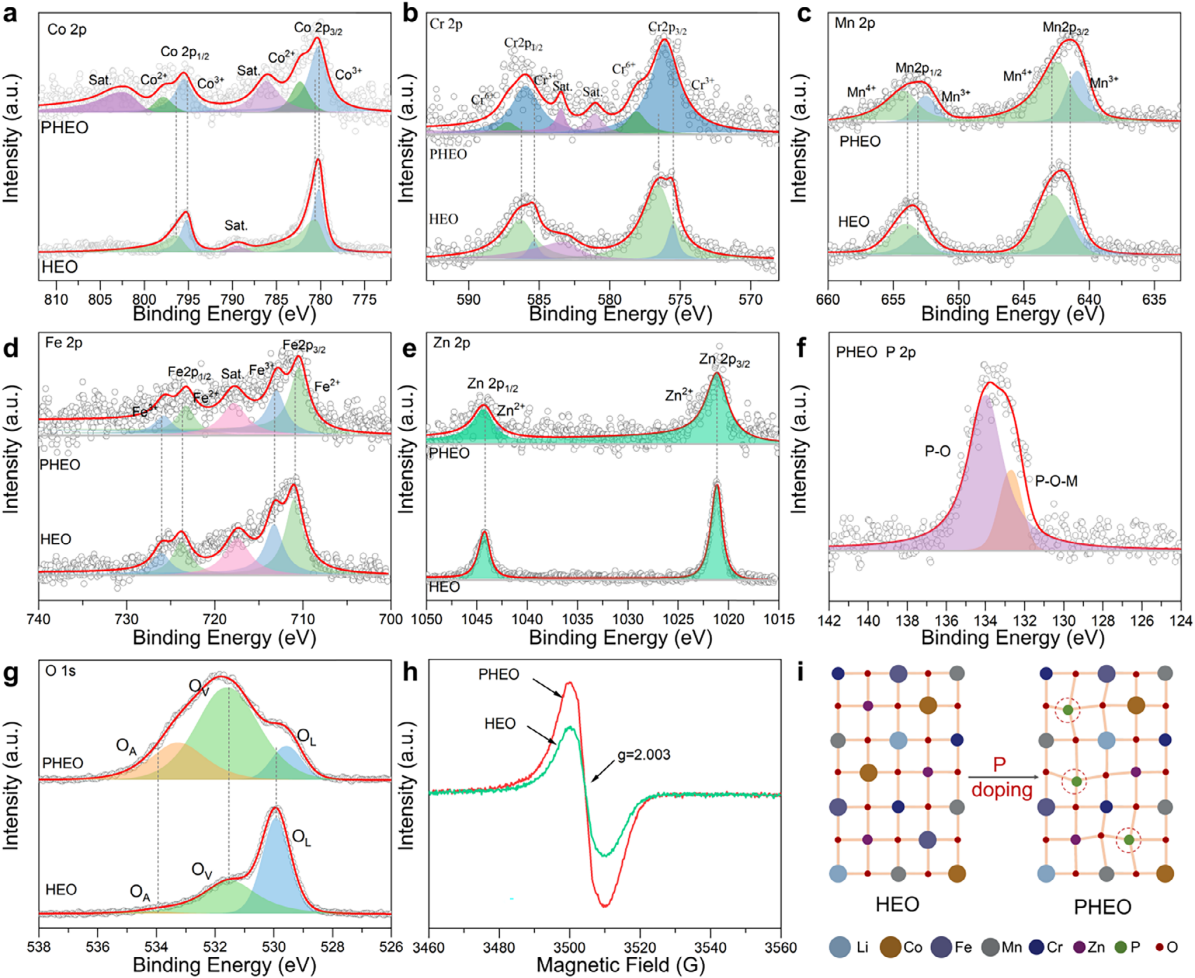
In‐depth surface chemical and structural characterization of HEO and PHEO. High‐resolution XPS core‐level spectra: a) Co 2p, b) Cr 2p, c) Mn 2p, d) Fe 2p, e) Zn 2p, f) P 2p, and g) O 1s, revealing chemical states and bonding environments. h) EPR spectra probe unpaired electron species and defect states. i) Schematic illustrations depicting the proposed chemical structures of HEO and PHEO.

XPS was employed to investigate the surface composition, elemental valence states, and the regulatory influence of P doping on the coordination environment of metal cations in HEO and PHEO. The survey spectrum (Figure S6, Supporting Information) shows distinct signals for Co, Cr, Mn, Fe, Zn, O, and P. After P doping, the Cr 2p and Co 2p peaks obviously shift toward higher binding energy, whereas Mn 2p and Fe 2p peaks shift toward lower binding energy, indicating significant electronic structure modulation.^[^ ^27^ ^]^ Specifically, as shown in **Figure** **2**a–e, the Co 2p_3/2_ peaks shift from 780.13 and 780.58 eV (assigned to Co^3+^ and Co^2+^) to 780.27 and 782.82 eV. The Cr 2p_3/2_ peaks, located at 575.49 and 576.52 eV and corresponding to Cr^3+^ and Cr^6+^ in HEO, shift to 576.09 and 578.06 eV in PHEO. The Mn 2p_3/2_ peaks shift from 641.44 and 642.83 eV (Mn^3+^ and Mn^4+^) to 640.89 and 642.32 eV. The Fe 2p_3/2_ peaks at 710.96 and 713.24 eV (Fe^2+^ and Fe^3+^) move to 710.43 and 712.97 eV. The Zn 2p peaks remained nearly constant at ≈1021.12 and 1021.16 eV due to its stable Zn^2+^ state.^[^ ^19^ ^]^ P doping also leads to considerable changes in the valence state distribution of metal cations. Detailed compositional comparisons are provided in Figure S7 and Table S3 (Supporting Information). Notably, the Cr^6+^ content decreases sharply from 84.68% to 14.28%, while the Co^3+^ content increases from 48.27% to 73.56%. The increase of metal states with free 3d electrons can efficiently enhance the electronic conductivity and rate performance.^[^ ^2^ ^]^ Although Fe and Mn valence states are also affected, the changes are less pronounced than those for Cr and Co.

The P 2p spectrum exhibited two peaks at 132.64 and 133.93 eV (Figure 2f), attributed to the P─O (PO_4_^3−^) and P─O─M bonds, respectively.^[^ ^28^ ^]^ They are generally considered to stabilize the framework structure of nanomaterials and enhance electron connectivity.^[^ ^29^ ^]^ This is attributed to the metal‐like character of P and its ability to form covalent bonds with surrounding atoms. These covalent bonds allow for tighter packing within the crystal structure, thereby suppressing the easy disruption of the spinel structure.^[^ ^22^ ^]^ High‐resolution O 1s spectra were deconvoluted into adsorbed oxygen (O_A_), O_V_, and lattice oxygen (O_L_),^[^ ^22^ ^]^ respectively (Figure 2g). O_V_ content increased from 41.77% in HEO to 64.01% in PHEO. While Li^+^ doping mainly generates O_V_ in HEO, in PHEO both Li^+^ and P doping synergistically enhance vacancy concentration. This is further supported by EPR results (Figure 2h), where PHEO shows a stronger O_V_ signal at g = 2.000. The increased O_V_ concentration is particularly beneficial for electrode materials as it promotes ion adsorption and provides more active sites for surface‐mediated charge storage.

**Figure 3:**
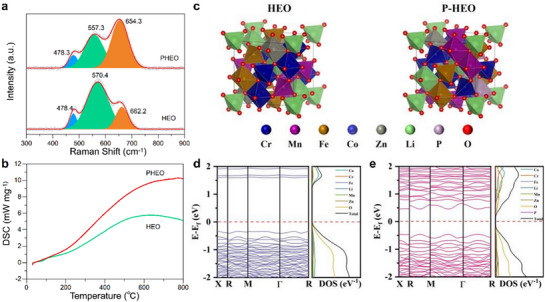
Comparative physicochemical property analysis of HEO and PHEO. a) Raman spectra identify vibrational modes and structural signatures. b) DSC curves assessing thermal behavior and stability. c) Proposed structural models and d) calculated electronic band structures with corresponding DOS profiles, elucidating electronic properties.

Raman spectroscopy also confirmed the spinel structure in both materials, with characteristic 3F_2g_ and A_1g_ vibrational modes. The typical characteristic peaks locate at 478.4, 570.4, 662.2 cm^−1^ for HEO and 478.3, 557.3, 654.3 cm^−1^ for PHEO (**Figure** **3**a). The frequency and intensity of the characteristic peaks are highly correlated with the cations in octahedral and tetrahedral sites and the symmetry of the crystal structure. The redshift of the A_1g_ mode in PHEO suggests structural defects introduction such as O_V_ induced by P doping.^[^ ^30^ ^]^ Differential scanning calorimetry (DSC) results (Figure 3b) showed that PHEO exhibited a stable exothermic profile from room temperature to 800 °C, while HEO displayed an exothermic process followed by a mild endothermic transition between 600 and 800 °C, likely related to the reduction of metal cations like Cr^3+^, Mn^3+^, Fe^3+^, and Co^3+^. The absence of this endothermic effect in PHEO further highlights the lattice‐stabilizing role of P incorporation,^[^ ^22^ ^]^ attributed to the introduction of PO_4_^3−^ groups that reinforce the crystal framework. Besides, density functional theory (DFT) calculations (Figure 3d) reveal a significant bandgap reduction from 1.23 eV in HEO to 0.30 eV in PHEO, facilitating easier electron transition from the valence to conduction band and thus higher conductivity. This result is attributed to the modulation of the electronic structure by P doping that induces more O_V_ sites and free electrons surrounding the metallic sites. Therefore, PHEO's improved electronic properties and enhanced structural stability contribute to its higher electronic conductivity, better reaction reversibility, and superior cycling stability as an anode material.

### 2.2 Electrochemical Performance

**Figure 4:**
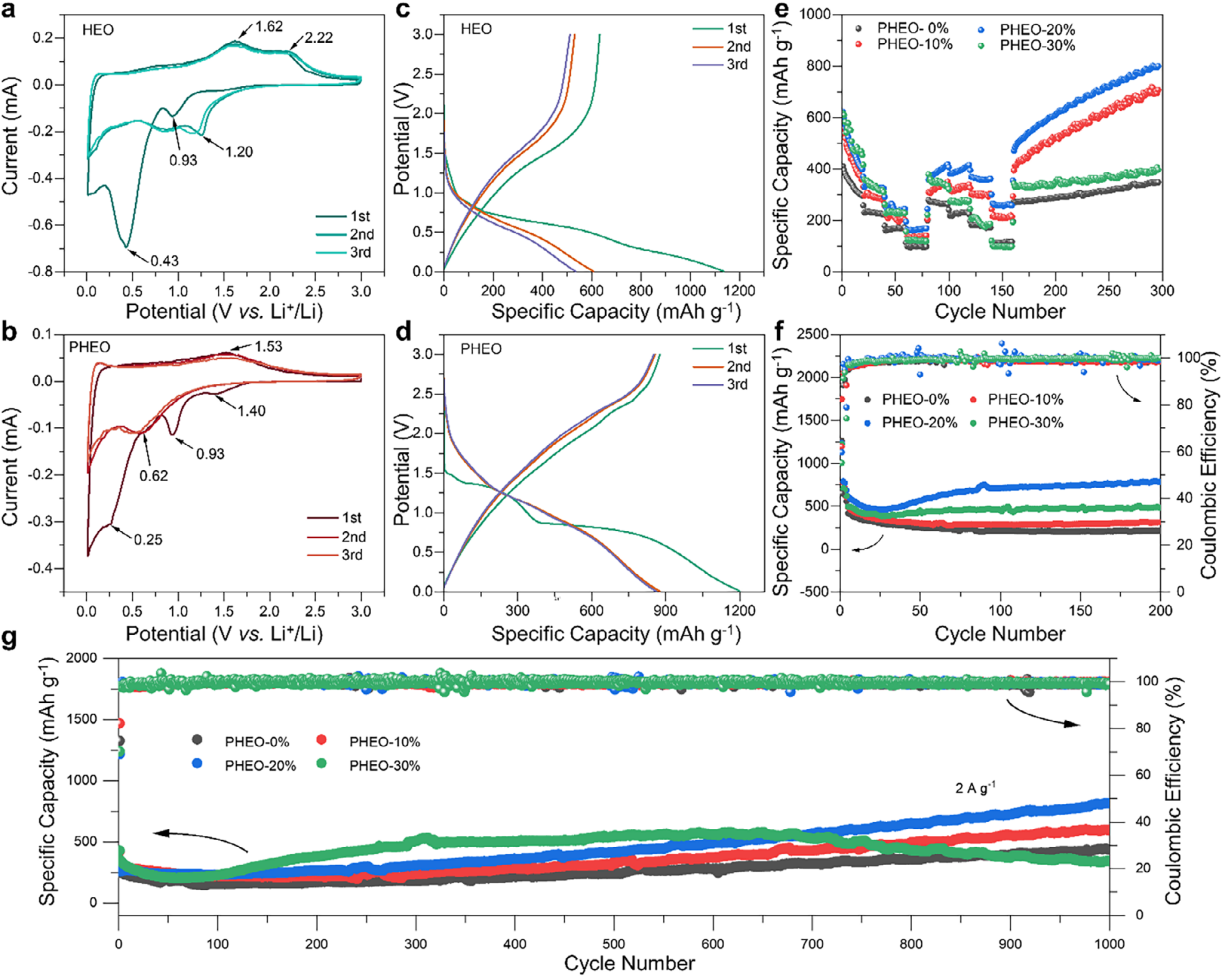
Comprehensive evaluation of electrochemical performance for HEO and PHEO anodes. a) First three CV curves of HEO at 0.1 mV s^−1^. b) First three GCD profiles of HEO at 0.5 A g^−1^. c) First three CV curves of PHEO at 0.1 mV s^−1^. d) First three GCD profiles of PHEO at 0.5 A g^−1^. e) Rate capability (measured at 0.5, 1, 2, and 5 A g^−1^) and long‐term cycling stability at 0.5 A g^−1^. Cycling performance and corresponding Coulombic efficiency of PHEO at f) 0.5 A g^−1^ and g) 2 A g^−1^.

The electrochemical properties of HEO and PHEO were evaluated using CR2032 coin‐type half‐cells. **Figure** **4**a,b present the initial three cyclic voltammetry (CV) curves for the HEO and PHEO anode materials, respectively. For HEO, the integrated area of the first‐cycle CV curve exceeds those of the subsequent two cycles, mainly due to irreversible side reactions such as solid electrolyte interphase (SEI) formation. In the first discharge, the reduction peak at 0.93 V corresponds to Li^+^ intercalation and the conversion of transition metal oxides (M_x_O_y_) into metallic M and Li_2_O: M_x_O_y_ + 2yLi^+^ + 2ye^−^ → xM + yLi_2_O. A secondary, irreversible peak near 0.43 V is associated with electrolyte decomposition and SEI growth.^[^ ^31^ ^]^ During the subsequent first charge, the oxidation peaks at 1.62 and 2.22 V are possibly attributed to the reverse delithiation process: xM + yLi_2_O → M_x_O_y_ + 2yLi^+^ + 2ye^−^. From the second cycle onward, the primary reduction peak shifts positively from 0.93 to 1.2 V (vs. Li^+^/Li), while the oxidation peak potential remains constant. This positive shift in reduction potential indicates a lower thermodynamic barrier for Li^+^ insertion, alongside stable thermodynamics and kinetics for Li^+^ extraction. The close overlap of the CV curves from the second and third cycles further confirms reversible reactions, low polarization, and efficient charge transfer kinetics. In contrast, PHEO exhibits a distinct first‐cycle CV profile. An initial reduction peak emerges at 1.40 V, which may be attributed to unidentified species or transient states formed during the synthesis of the high‐entropy material. Such a phenomenon is also observed in other high‐entropy systems.^[^ ^32^ ^]^ A second reduction peak at ≈0.93 V corresponds to the Li^+^ intercalation, followed by an irreversible peak near 0.25 V associated with SEI formation. In subsequent cycles, the main reduction peak shifts negatively from 0.93 to 0.62 V, and the oxidation peak also moves to a lower potential compared to that of HEO. These shifts suggest that P doping effectively modulates the local chemical environment of the oxide matrix and induces a fundamentally different Li^+^ storage mechanism relative to the undoped HEO.^[^ ^33^ ^]^

Figure 4c,d present the first three galvanostatic charge/discharge (GCD) profiles of HEO and PHEO measured at 0.2 A·g^−1^ within the voltage window of 0.01–3 V. Both electrodes exhibit two distinct discharge plateaus: a lower‐potential plateau corresponding to irreversible SEI formation, and a higher‐potential plateau ascribed to the conversion of M_x_O_y_ to metallic species and/or the Li^+^ intercalation.^[^ ^34^ ^]^ Quantitatively, HEO delivers specific capacities of 1136.7, 606.5, and 534.5 mAh g^−1^ over the initial three cycles, whereas PHEO achieves 1200.7, 876.3, and 860.6 mAh g^−1^.^[^ ^22^ ^]^ These values align with the involvement of multiple redox‐active couples, including Cr^3+^/Cr^6+^, Mn^3+^/Mn^4+^, Fe^2+^/Fe^3+^, and Co^3+^/Co^3+^, during the electrochemical processes, as reported in previous studies.^[^ ^35^, ^36^ ^]^ It can be inferred that, in addition to the direct contribution of P doping to capacity enhancement, the performance difference between PHEO and HEO may also originate from the notable modifications in the local microstructure and chemical states of Cr^3+^/Cr^6+^ and Co^3+^/Co^3+^, as evidenced by earlier structural and spectroscopic analysis.

Rate capability evaluations of HEO and PHEO are shown in Figure 4e. At current densities of 0.5, 1, 2, and 5 A g^−1^, HEO delivers 264.6, 228.4, 172.6, and 117.0 mAh g^−1^, respectively, whereas PHEO reaches 417.1, 415.0, 361.7, and 259.0 mAh g^−1^. When the current density is restored to 0.5 A g^−1^, PHEO rapidly recovers to 471.4 mAh g^−1^ and gradually rises to ≈800 mAh g^−1^ by the 300th cycle. Under a constant current of 0.5 A g^−1^ (Figure 4f), PHEO‐20% delivers an initial capacity of 686.2 mAh g^−1^ compared to 556.5 mAh g^−1^ for HEO. Pronounced activation behavior in PHEO leads to a capacity of 798.8 mAh g^−1^ after 200 cycles (116.4% retention), in contrast to HEO, which retains only 39.3% retention. The first‐cycle Coulombic efficiency is also higher for PHEO (73.0% vs. 53.4%). At 2 A g^−1^ (Figure 4g), PHEO‐20% achieves 817.2 mAh g^−1^ after 1000 cycles (149.6% retention), while HEO attains only 442.7 mAh g^−1^ (54% of PHEO‐20%). The progressive capacity increase is likely attributable to cycling‐induced grain refinement, which enlarges the electrode–electrolyte contact area and exposes additional active sites.^[^ ^12^, ^19^ ^]^ Moreover, the outstanding cycling performance of PHEO can be attributed to the role of PO_4_^3−^ units as key structural stabilizers, which act synergistically with the high‐entropy effect of multiple cations to enhance the overall stability of the material. However, an anomalous behavior is observed in PHEO‐30%, which fails to maintain the enhanced capacity beyond ≈600 cycles. This may be ascribed to the presence of non‐spinel impurities, as identified in the structural analysis. It can be hypothesized that under prolonged high‐rate cycling, mechanical stress accumulates around these impurities, inducing gradual structural degradation. Although the structural stability is initially afforded by PO_4_^3^^−^ units and the high‐entropy effect, the detrimental impact of impurities eventually outweighs these stabilizing factors upon prolonged cycling, resulting in the observed capacity fading.

**Figure 5:**
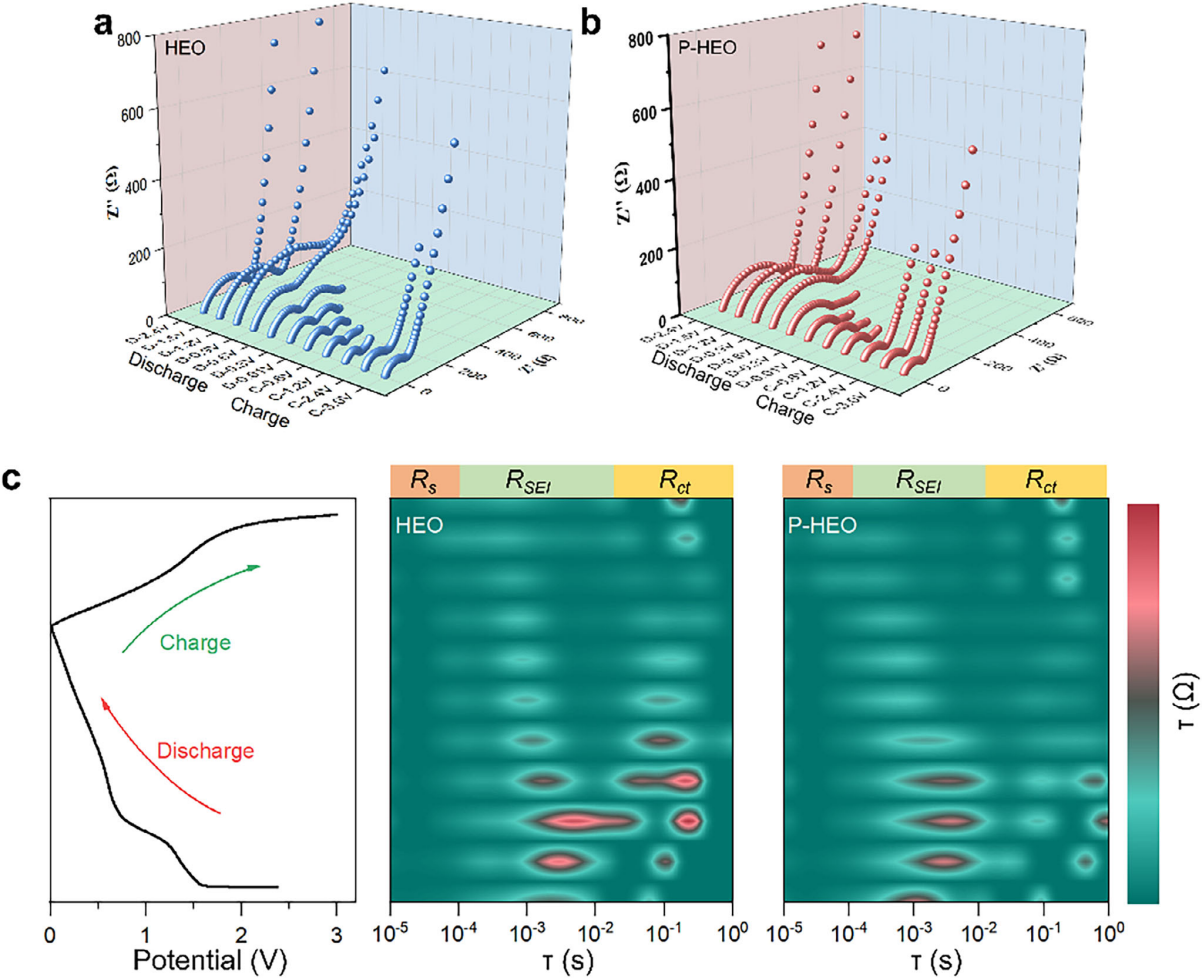
Operando EIS and relaxation analysis. 3D in situ EIS mapping during the first galvanostatic cycle for a) HEO and b) PHEO, visualizing impedance evolution. c) Calculated DRT plots derived from the in situ EIS data, resolving distinct electrochemical processes.

To elucidate the electrochemical behavior of HEO and PHEO, in situ electrochemical impedance spectroscopy (EIS) was performed to monitor Li^+^ and electron diffusion resistance during cycling (Figure S8, Supporting Information). Throughout the first charge/discharge cycle, the charge‐transfer resistance (*R_ct_*) of PHEO remains comparable to or lower than that of HEO, indicating enhanced charge‐transfer kinetics. Below 0.6 V, both electrodes exhibit two semicircles: the left corresponding to SEI‐related resistance (*R_SEI_*) and the right to charge‐transfer processes (*R_ct_*). In this voltage range, *R_SEI_* dominates the overall impedance. PHEO consistently displays higher *R_SEI_* but lower *R_ct_* than HEO, suggesting the formation of a more resistive yet structurally stable SEI layer. 3D in situ EIS plots (**Figure** **5**a,b) track the impedance evolution during discharge/charge, providing insight into Li^+^ diffusion kinetics. In the early stage of discharge (2.4–0.9 V), both *R_ct_* and Warburg impedance remain relatively high. For HEO, the diffusion impedance initially increases, likely due to Li^+^ accumulation hindering charge‐transfer, before subsequently decreasing. In contrast, PHEO maintains more stable impedance behavior, attributable to its richer ion diffusion pathways. As discharge deepens (0.9–0.01V), *R_ct_* progressively decreases, consistent with the sharp reduction peak observed in the first cycle. The irreversible capacity loss during this stage is primarily attributed to Li^+^ consumption from SEI formation. Upon charging to 1.2 V, impedance further declines as metallic species react with Li_2_O and surface‐active materials reorganize into Li_x_MO_y_ intermediates. Delithiation of Li_x_MO_y_ occurs only above 2.4 V, where HEO exhibits a slight increase in *R_ct_* due to reconversion to oxides, whereas PHEO sustains lower diffusion resistance.

Furthermore, the distribution of relaxation times (DRT) analysis was applied to resolve the impedance evolution. Unlike conventional equivalent circuit modeling, DRT does not require predefinition of a circuit topology, thereby minimizing potential biases and errors from inappropriate assumptions.^[^ ^37^ ^]^ As shown in Figure 5c, three main relaxation time regions are identified: ohmic resistance (*R_s_*, 10^−5^–10^−4^ s), SEI‐related resistance (*R_SEI_*, 10^−4^–10^−1^ s), and charge‐transfer resistance (*R_ct_*, 10^−1^‐10^0^ s). The most notable differences between HEO and PHEO lie in the *R_SEI_* and *R_ct_* regions, indicating that P doping contributes to SEI stabilization, mitigates volume‐change‐induced fracture/reconstruction, and maintains a robust electrode–electrolyte interface. This structural integrity, combined with improved electronic conductivity, underpins the superior ion and electron transport properties of PHEO.

**Figure 6:**
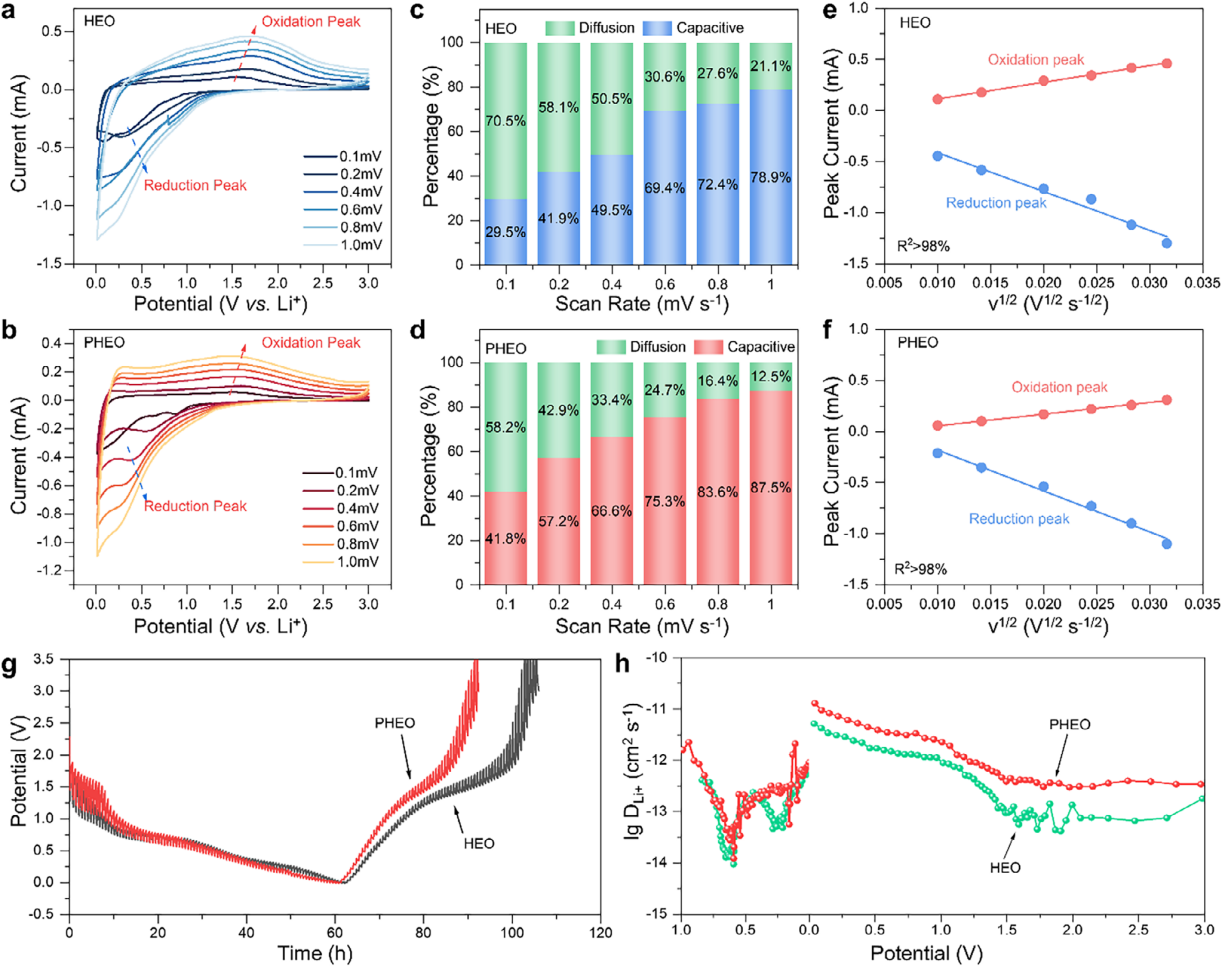
Kinetic analysis of lithium‐ion storage behavior. CV curves of a) HEO and b) PHEO at varying scan rates (0.1 to 1.0 mV s^−1^). Bar chart summarizing the ratios of capacitive contribution at different scan rates for c) HEO and d) PHEO. Linear fitting of *I_p_* vs. *v^1/2^* for e) HEO and f) PHEO. g) GITT profiles and h) calculated apparent Li^+^ diffusion coefficients for both materials.

**Figure** **6**a,b compares the CV curves of HEO and PHEO at various scan rates (*ν*), which reflect Li^+^ diffusion kinetics. In a reversible system, the peak current (*I_p_*) follows the Randles–Sevcik equation at 25 °C:^[^ ^38^, ^39^ ^]^

(1) $$I_{p}=\left(2.69\times 10^{5}\right)n^{\frac{3}{2}}AD_{Li^{+}}^{\frac{1}{2}}Cv^{\frac{1}{2}}$$

(2) $$D_{Li^{+}}={\left(\frac{I_{p}}{v^{\frac{1}{2}}}\right)}^{2}\frac{1}{{\left(2.69\times 10^{5}\right)}^{2}n^{3}A^{2}C^{2}}$$

where *n* is the number of electrons transferred, *A* the electrode area (cm^2^), $D_{Li^{+}}$ the Li^+^ diffusion coefficient (cm^2^ s^−1^), *C* the concentration of Li^+^ carriers (mol cm^−3^), *ν* the scan rate (V s^−1^), and *I_p_* the peak current (A). The slope (*b*‐value) from log(*I_p_*)‐log(*ν*) fitting was 0.45/0.63 for HEO and 0.70/0.72 for PHEO (Figure S9, Supporting Information), indicating a larger pseudocapacitive contribution in PHEO. To quantify this effect, capacitive contribution analysis was systematically performed across scan rates from 0.1 to 1 mV s^−1^ (Figure 6c,d). The pseudocapacitive contribution is consistently more pronounced in PHEO than in HEO at all scan rates. Specifically, at 0.1 and 1 mV s^−1^ (Figure S10, Supporting Information), the pseudocapacitive fractions reached 41.8% and 87.5%, respectively. The two values are significantly higher than those of HEO. This enhancement arises from: 1) P doping‐induced grain refinement and increased specific surface area; and 2) abundant surface and bulk defects providing more active sites for Li⁺ adsorption. This elevated pseudocapacitance facilitates faster Li⁺ insertion/extraction, thereby sustaining high‐rate performance and capacity retention.

Linear fitting was used to estimate the overall Li^+^ diffusion coefficients during insertion and extraction (Figure 6e,f), revealing that $D_{Li^{+}}$ in PHEO is approximately twice that in HEO (Table S4, Supporting Information). To further quantify $D_{Li^{+}}$, galvanostatic intermittent titration technique (GITT) was applied. The results are presented in Figure 6g,h and Figure S11 (Supporting Information). During lithiation, $D_{Li^{+}}$ values range from 10^−14^ to 10^−11.5^ cm^2^ s^−1^ for PHEO and HEO, with PHEO consistently exhibiting higher values. During delithiation, PHEO again surpasses HEO, with $D_{Li^{+}}$ values of 10^−12.5^–10^−11^ cm^2^ s^−1^ compared to 10^−13.5^–10^−11.5^ cm^2^ s^−1^ for HEO. The highest diffusion coefficients are observed at deep lithiation near ≈0 V, consistent with EIS results showing minimal *R_ct_* under these conditions.

### 2.3 Li^+^ Storage Mechanism

**Figure 7:**
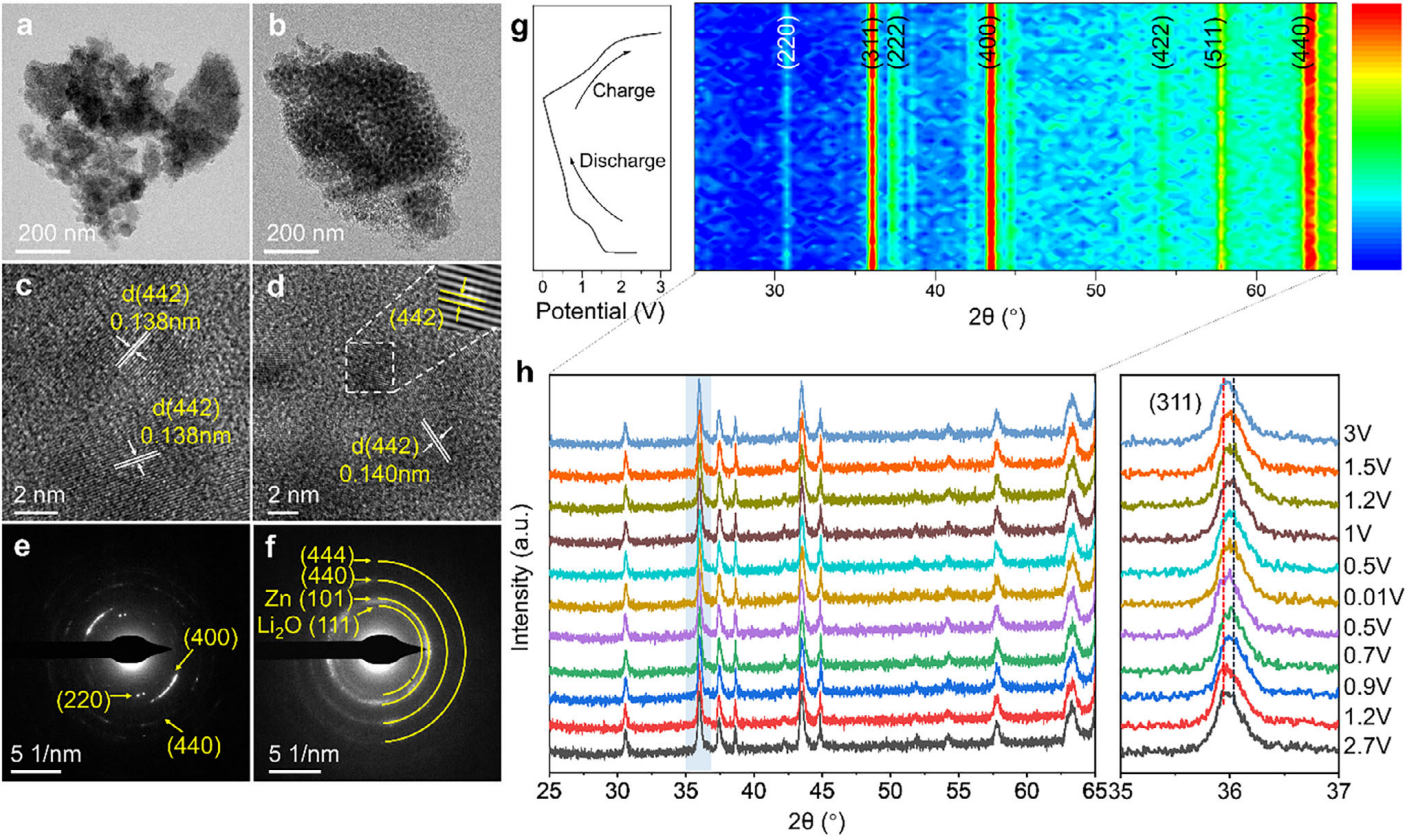
Structural evolution analysis during electrochemical cycling. Post‐mortem TEM images of a) PHEO and b) HEO after the first cycle. High‐resolution TEM images of c) PHEO and d) HEO after the first cycle. SAED patterns of cycled e) PHEO and f) HEO. g) Ex situ XRD patterns of PHEO acquired throughout the first charge/discharge cycle, monitoring phase changes in real‐time.

To elucidate the electrochemical lithium storage mechanisms of HEO and PHEO, TEM characterization was performed on the electrode after the first cycle (**Figure** **7**a–d). HRTEM analysis revealed that HEO predominantly exhibited lattice fringes corresponding to the (442) plane of the *Fd3m* spinel structure, along with the presence of a Li_2_O phase. Li_2_O originates from the conversion reaction M_x_O_y_ + 2yLi → xM + yLi_2_O during discharge.^[^ ^40^ ^]^ Due to incomplete reversibility, a portion of Li_2_O remained after charge. In contrast, no Li_2_O was detected in HRTEM images of PHEO. SAED results further support this observation. For HEO, distinct diffraction spots were observed for the Zn metal phase (101) plane and the (111) plane of nanocrystalline Li_2_O,^[^ ^18^ ^]^ whereas PHEO showed only spinel diffraction spots representing to the (220), (440), and (442) planes. These results indicate that HEO undergoes a conversion‐type lithium storage, while PHEO demonstrates a different Li^+^ storage mechanism with superior reversibility, an advantageous trait for long‐term electrochemical performance.

To further investigate the Li^+^ storage mechanism of the PHEO, ex situ XRD was conducted during the first cycle (Figure 7g). At the open‐circuit voltage, the PHEO electrode exhibited well‐defined peaks for the (220), (311), (222), (400), (511), and (440) planes. Upon discharging to 0.01 V and subsequently charging to 3 V, these diffraction peaks showed no significant changes in intensity or disappearance. A slight shift in the main peak was observed at different states of charge. As shown in the enlarged (311) peak profile (Figure 7h), it moves to higher angle with the Li^+^ insertion and re‐shifts to lower angle after Li^+^ extraction, which may be attributed to the high binding between inserted Li^+^ and anions, thus causing lattice distortion.^[^ ^22^ ^]^ This structural invariance confirms that PHEO maintains a zero‐strain structural framework in Li^+^ storage process.^[^ ^41^ ^]^ Computational simulations also support the zero‐strain characteristics of PHEO. The cell dimension relative to the initial state shows a volume expansion with +0.74% during charging and a contraction of ‐0.11% during discharging, resulting in a net volume change of only 0.85%. Therefore, both experimental and simulation results thus verify the stable chemical structure of PHEO, which underpins its outstanding electrochemical performance during cycling.

Based on the combined results, the superior electrochemical activity and cycling stability of PHEO can be attributed to a suite of structural and electronic advantages conferred by P doping. The high electronegativity of P increases the number of electrochemically active lithium storage sites and facilitates more accessible pathways for Li^+^ insertion and extraction. In addition, the high concentration of O_V_ introduced by doping creates abundant channels for rapid Li^+^ transport, significantly enhancing ionic diffusion kinetics. Furthermore, P‐induced lattice distortion modulates the electronic structure of the spinel framework, thereby improving its intrinsic Li^+^ storage activity. These beneficial effects are synergistically reinforced by the robust covalent nature of P─O bonds and the entropy stabilization inherent to the high‐entropy system. Together, these features endow PHEO with exceptional structural integrity and sustained electrochemical performance over extended cycling.

## 3 Conclusion

In this work, HEO and PHEO anode materials were successfully synthesized via a sol–gel method and systematically evaluated for LIB applications. PHEO retains the spinel framework while exhibiting reduced particle sizes, improved dispersion, and an optimized electronic configuration, resulting from the incorporation of stable PO_4_^3−^ groups, modulation of metal valence states, and increased O_V_ content. These structural and electronic modifications significantly enhance Li^+^ storage activity, ion diffusion kinetics, and structural stability. Electrochemically, PHEO demonstrates high capacity, excellent rate capability, and outstanding cycling performance, achieving 686.2 mAh g^−1^ at 0.5 A g^−1^ and maintaining 149.6% of its capacity after 1000 cycles at 2 A g^−1^. Mechanistic studies reveal that while HEO undergoes a conversion‐type lithium storage mechanism, PHEO exhibits a fundamentally different process with a dominated role of surface pseudocapacitance and near‐zero‐strain characteristics, enabling faster kinetics and superior reversibility. These findings establish PHEO as a highly promising anode material for high‐performance LIBs.

## 4 Experimental Section

### Chemicals

Chemicals used for high‐entropy oxides preparation include lithium nitrate (LiNO_3_, 99.9%, Aladdin), chromium(III) nitrate nonahydrate (Cr(NO_3_)_3_·9H_2_O, 99.9%, Aladdin), manganese(II) nitrate hexahydrate (Mn(NO_3_)_2_·6H_2_O, 99.9%, Aladdin), iron(III) nitrate nonahydrate (Fe(NO_3_)_3_·9H_2_O, 99.9%, Aladdin), cobalt(II) nitrate hexahydrate (Co(NO_3_)_2_·6H_2_O, 99.9%, Aladdin), and copper(II) nitrate hexahydrate (ZnCl_2_, 99.9%, Aladdin) were used as metal sources. Citric acid (C_6_H_8_O_7_, ≥99.5%, Aladdin) was employed as the complexing agent. Ammonium dihydrogen phosphate (NH_4_H_2_PO_4_, ≥99%, Aladdin) was used as phosphorus source. For electrode preparation, conductive graphite (Super P, Timcal), polyvinylidene fluoride (PVDF, Arkema) as the binder, and N‐methyl‐2‐pyrrolidone (NMP, 99.5%, Aladdin) as the solvent were utilized. The electrolyte components included ethylene carbonate (EC, 99.9%, Aladdin), methyl carbonate (EMC, 99.9%, Aladdin), and dimethyl carbonate (DMC, 99.9%, Aladdin), which were mixed to form the organic electrolyte solution. All chemicals were used as received without further purification.

### Synthesis of [P_x_(LiCrMnFeCoZn)_1‐x_]_3_O_4_

The high‐entropy [P_x_(LiCrMnFeCoZn)_1‐x_]_3_O_4_ anode material was synthesized via a sol–gel method. Metal nitrates including LiNO_3_, Cr(NO_3_)_3_·9H_2_O, Mn(NO_3_)_2_·6H_2_O, Fe(NO_3_)_3_·9H_2_O, Co(NO_3_)_2_·6H_2_O, and ZnCl_2_ were dissolved in 100 mL deionized water according to the stoichiometric ratios of metal elements (Table S1, Supporting Information) to prepare a homogeneous mixed salt solution with a concentration of 0.01 mol L^−1^. After magnetic stirring for 30 min to obtain a clear and uniform solution, NH_4_H_2_PO_4_ was added as the phosphorus source in a predetermined proportion, followed by another 30 min of magnetic stirring. Subsequently, anhydrous citric acid was introduced and the same procedure was repeated. The solution was then heated to 70 °C with continuous stirring until a brown viscous gel formed. The obtained gel was thoroughly dried in an oven at 120 °C to yield a loose powder, which was subsequently ground into fine particles and calcined in a tube furnace at 750 °C for 2 h. Finally, the sample powder was further ground and stored under dry conditions.

### Electrode Preparation

The lithium storage performance of the high‐entropy metal oxide anode was evaluated using CR2032 coin cells. The electrode slurry was prepared by uniformly mixing the active material (70 wt.%), conductive graphite (20 wt.%), and PVDF (10 wt.%) in NMP solvent. The slurry was then coated onto a copper current collector and dried in a vacuum oven at 80 °C for 12 h to fabricate the anode electrode. After compaction, the electrode film was cut into circular discs with a diameter of 12 mm, with an active material mass loading of ≈1.0 mg·cm^−2^. The coin cells were assembled in an Ar‐filled glove box with both H_2_O and O_2_ contents maintained below 0.1 ppm. The electrolyte consisted of 1.0 mol L^−1^ LiPF_6_ dissolved in a mixture of EC, EMC, and DMC (volume ratio = 1:1:1). Lithium metal foil and Whatman GF/D glass fiber separator (19 mm in diameter) were employed as the counter electrode and separator, respectively.

### Material Characterization

X‐ray diffraction (XRD) measurements, including ex situ analyses, were conducted on a Bruker D8 Advance X‐ray diffractometer with Cu Kα radiation to determine the phase composition of the materials. The morphology, particle size, and elemental distribution of the synthesized samples were characterized using field‐emission scanning electron microscopy (FE‐SEM, Thermo Fisher Scientific Apreo 2 S Lovac). Microstructural features such as lattice fringes and elemental mapping were examined by transmission electron microscopy (TEM, Thermo Fisher Scientific FEI Talos F200X), which also allowed observation of structural evolution during phase transitions. The surface chemical states of both HEO and PHEO were investigated by X‐ray photoelectron spectroscopy (XPS) on a Thermo Scientific Escalab Xi+ system. Raman spectroscopy was performed with a Renishaw inVia Qontor spectrometer (Renishaw, UK) using a 532 nm laser source to obtain molecular structural information. Elemental composition of the sample was quantified by inductively coupled plasma (ICP) analysis using a PerkinElmer 8300 instrument. Thermal behavior and stability were assessed using equipment from NETZSCH‐Gerätebau GmbH.

### DFT Calculation

All first‐principles calculations within the framework of density functional theory (DFT) were conducted employing the Vienna Ab Initio Simulation Package (VASP). The Perdew–Burke–Ernzerhof (PBE) functional under the generalized gradient approximation (GGA) was adopted to treat electron exchange and correlation interactions. A plane‐wave cutoff energy of 450 eV was used for the basis set. The chemical composition reflected actual synthesis conditions and was consistent with inductively coupled plasma (ICP) measurements. Spin polarization was incorporated by setting ISPIN = 2. To mitigate interactions between periodic images, a vacuum spacing of 20 Å was introduced. Structural relaxation was performed until the total energy and atomic forces converged to below 1 × 10^−5^ eV and 0.03 eV Å^−1^, respectively.

### Electrochemical Measurements

Electrode preparation: the obtained HEO active material 70 wt.%) was uniformly mixed with acetylene black (20 wt.%) and PVDF (polyvinylidene fluoride) (10 wt.%), then dissolved them in N‐methyl pyrrolidone (NMP) to form a slurry. Coat the slurry onto copper foil and bake it at 60 °C for 8 h. The dried electrode film was compacted and subsequently divided into small circular pieces with diameter of 12 mm. The amount of active material loaded onto each flake is ≈1.8–2.2 mg·cm^−2^. The electrochemical properties of the HEO anodes were tested with CR2032‐type button half‐cells. The half‐cells were constructed within a glove box filled with argon gas, ensuring the water and oxygen content remained below 1 ppm. The HEO was acted as the active material for anode, lithium plate (Φ15, 1.0 mm in thickness) was used as the counter electrode and reference electrode, and polypropylene (Celgard 2500) was used as the separator. The electrolyte (100 µl) used was a mixture of EC, EMC, and DEC in a 1:1:1 volume ratio with 1.0 mol·L^−1^ LiPF_6_. The performances of the batteries were tested using a LAND battery testing unit. The cyclic voltammogram (CV) tests were recorded on an electrochemical workstation (CH660E) with scanning voltage from 0.01 to 3V (vs. Li^+^/Li) at 0.1 mV·s^−1^. The electrochemical impedance spectroscopy (EIS) was conducted on an electrochemical workstation (VarsaSATA 3) across 10^5^ Hz to 10^−2^ Hz, with an amplitude of 5 mV.

Through the equivalent fitting calculation of the EIS curve, the impedance magnitudes of various components can be analyzed both qualitatively and quantitatively. The lithium‐ion diffusion coefficient at the electrode surface can be calculated using Equation:

(3) $$D_{Li^{+}}=\frac{1}{2}\frac{R^{2}T^{2}}{A^{2}n^{4}F^{4}C^{2}\sigma^{2}}$$

$D_{Li^{+}}$ is the diffusion coefficient of Li ions, R is the gas constant, T is the absolute temperature, A is the effective electrode area, n is the number of electrons transferred per molecule, F is the Faraday constant, C is the bulk concentration of charge carrier ions, σ is the Warburg coefficient, calculated from Equation:

(4) $$Z^{\prime }=R_{e}+R_{\mathrm{ct}}+\sigma \omega^{-\frac{1}{2}}$$

where Z′ is the real part of the impedance, ω is the angular frequency.

## Conflict of Interest

The authors declare no conflict of interest.

## Supporting information



Supporting Information

## Data Availability

The data that support the findings of this study are available in the supplementary material of this article.
