# Supplementary material for: Co‐Stabilization of High‐Entropy Oxides by Entropy and Polyanionic Units toward High‐Capacity Zero‐Strain Anodes for Advanced Lithium‐Ion Batteries
Source: Adv Sci (Weinh). 2025 Nov 8;13(5):e16795. doi: 10.1002/advs.202516795 (PMC12850283; doi:10.1002/advs.202516795)
Supplement: Supplementary file 1 — Supporting Information [file ADVS-13-e16795-s001.docx]

Supporting Information

Co-Stabilization of High-Entropy Oxides by Entropy and Polyanionic Units toward High-Capacity Zero-Strain Anodes for Advanced Lithium-Ion Batteries

Xiehang Chen^1^, Yang Xiang^2^, Cong Li^1^, Shipai Song^1^, Xincong Liu^1,3^, Bing Luo^1,*^ and Yong Xiang^1,2^

X. Chen, C. Li, S. Song, X. Liu, B. Luo, Y. Xiang

Frontier Center of Energy Distribution and Integration, Tianfu Jiangxi Lab, Huoju Avenue, Futian Sub-District, Jianyang city, Chengdu, Sichuan 641419, China.
E-mail: [luobing@jxl.ac.cn](mailto:luobing@jxl.ac.cn)

X. Chen, Y. Xiang, Y. Xiang
State Key Laboratory of Electronic Thin Films & Integrated Devices, School of Energy Science and Engineering, University of Electronic Science and Technology of China, 2006 Xiyuan Avenue, West High-Tech Zone, Chengdu, Sichuan 611731, China.

X. Liu

School of Architecture and Civil Engineering, Chengdu University, Chengdu, Sichuan, 610106, PR China


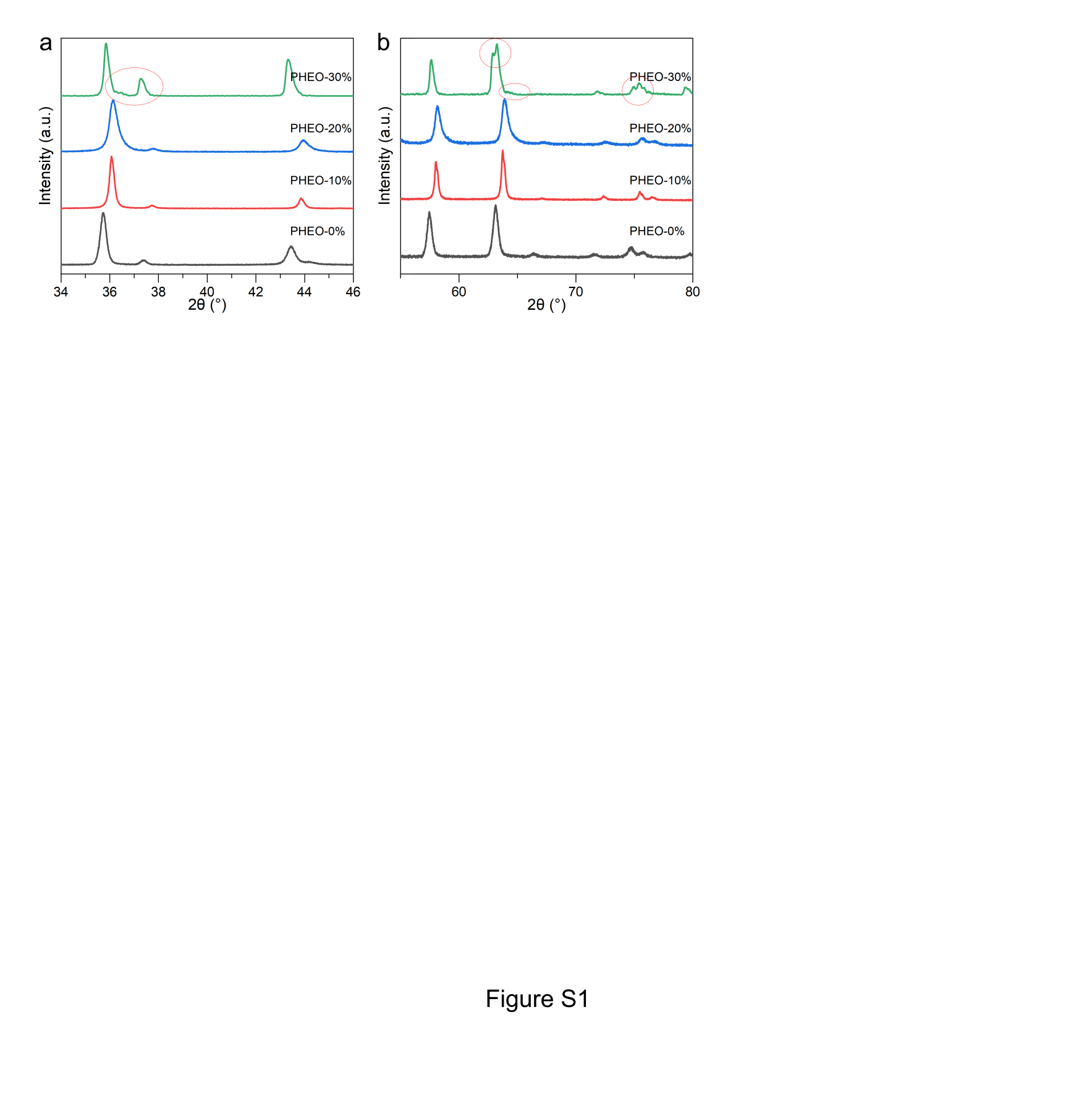


Figure S1. The enlarged XRD patterns of PHEO samples within (a) 34°~46° and (b) 55°~80°. In comparison to HEO, the extra signals are highlighted by red circles.


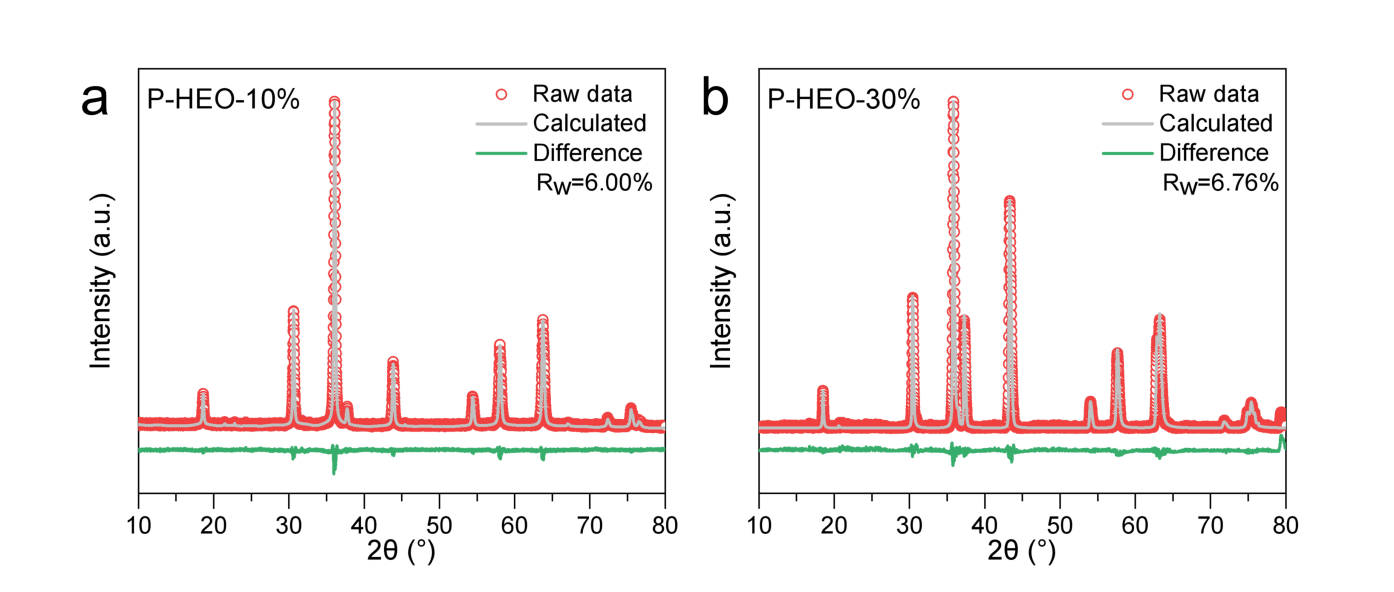


**Figure S2.** The Rietveld refinement results of PHEO-10% amd PHEO-30% XRD patterns.


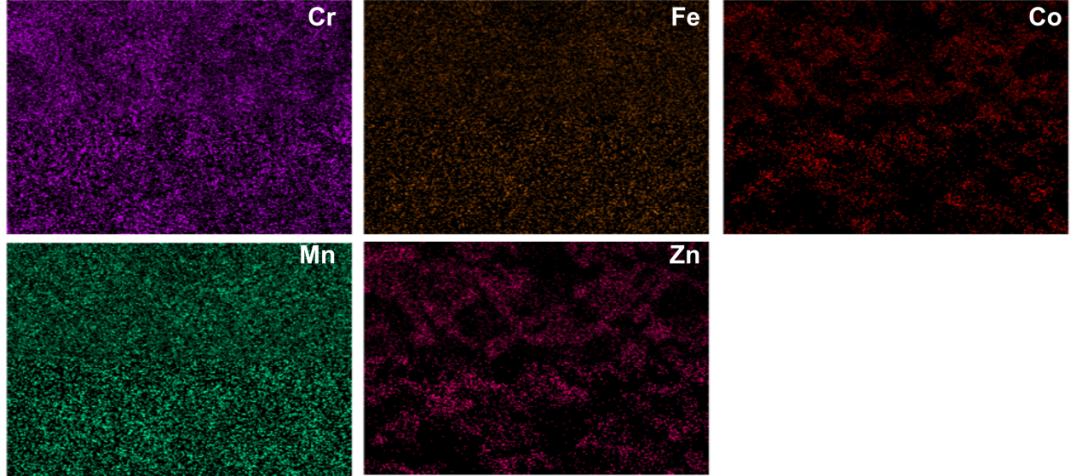


**Figure S3.** The SEM EDS mapping results of HEO.


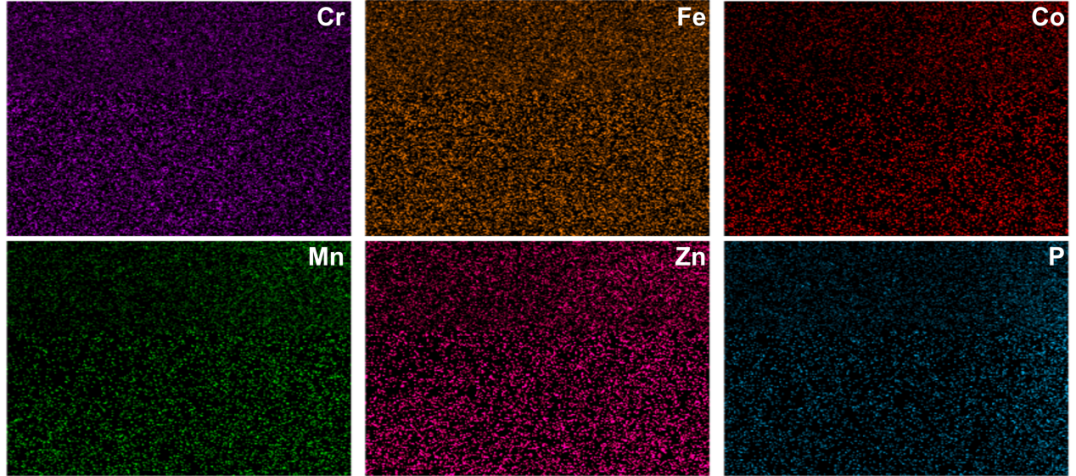


**Figure S4.** The SEM EDS mapping results of PHEO.


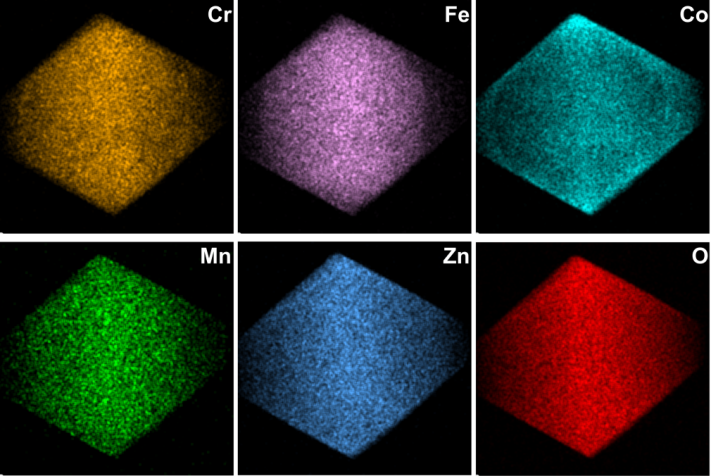


**Figure S5.** The TEM EDS mapping results of HEO.


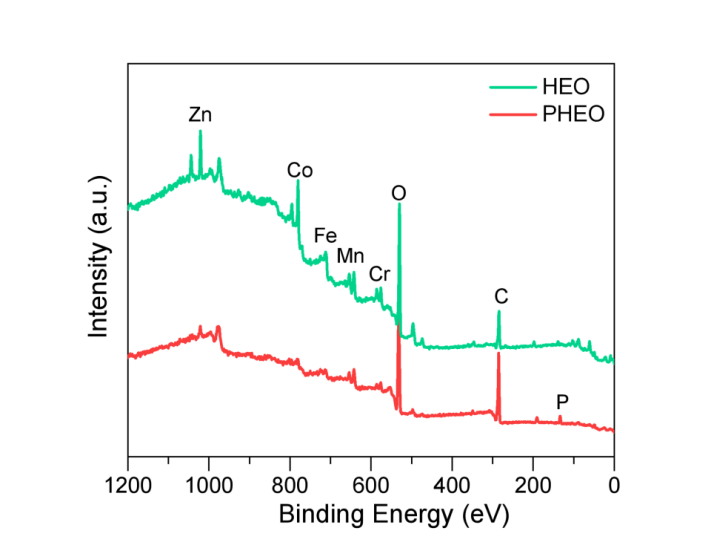


**Figure S6.** The XPS profiles of HEO and PHEO.


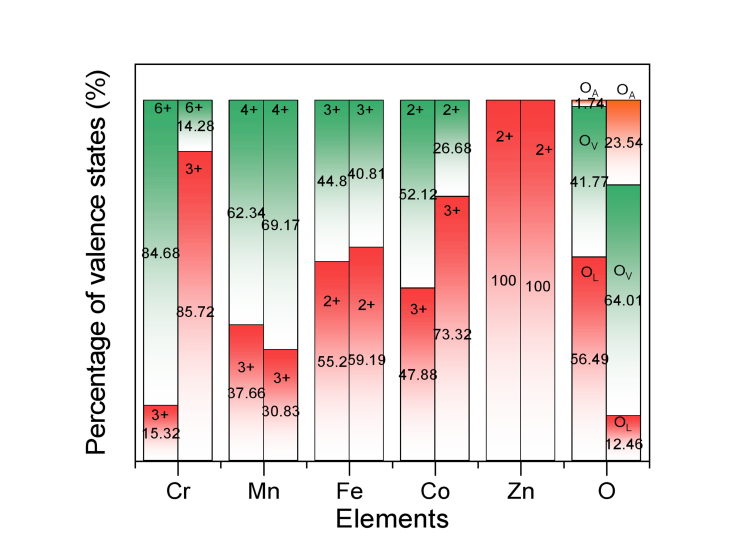


**Figure S7.** The percentage of valence states of different elements of HEO (left) and PHEO (right).


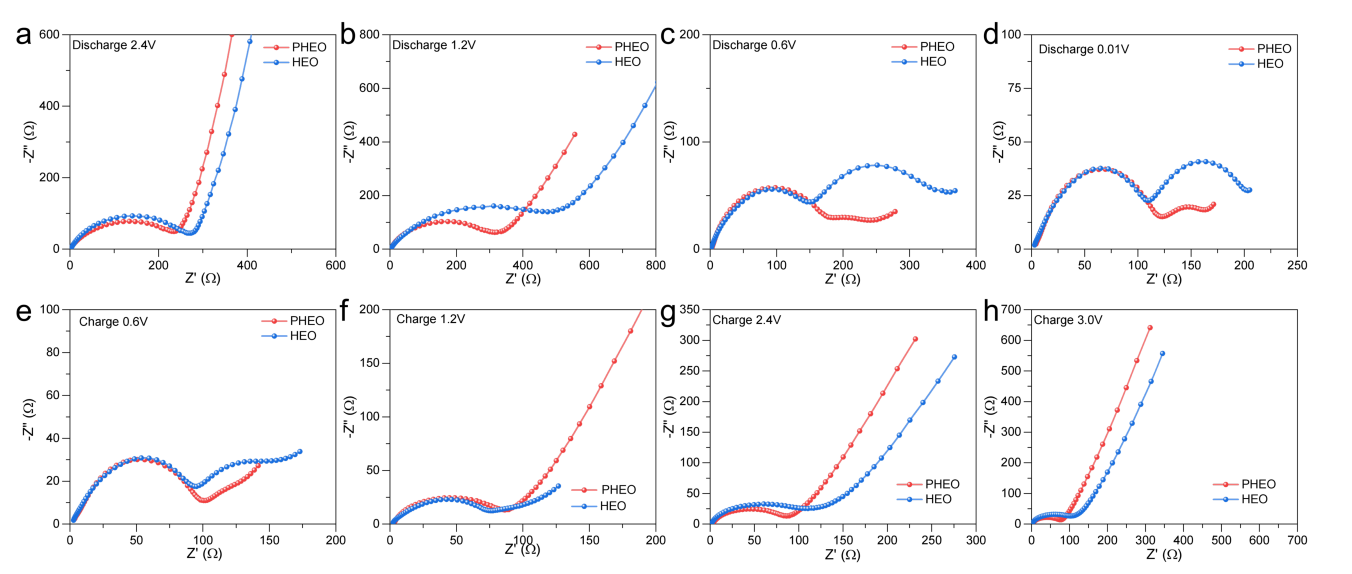


**Figure S8.** The Nyquist curves at different potentials in the first cycle of HEO and PHEO. (a-d) Discharge states: (a) 2.4 V, (b) 1.2 V, (c) 0.6 V, and (d) 0.01 V. (e-h) Charge states: (e) 0.6 V, (f) 1.2 V, (g) 2.4 V, and (h) 3.0 V.


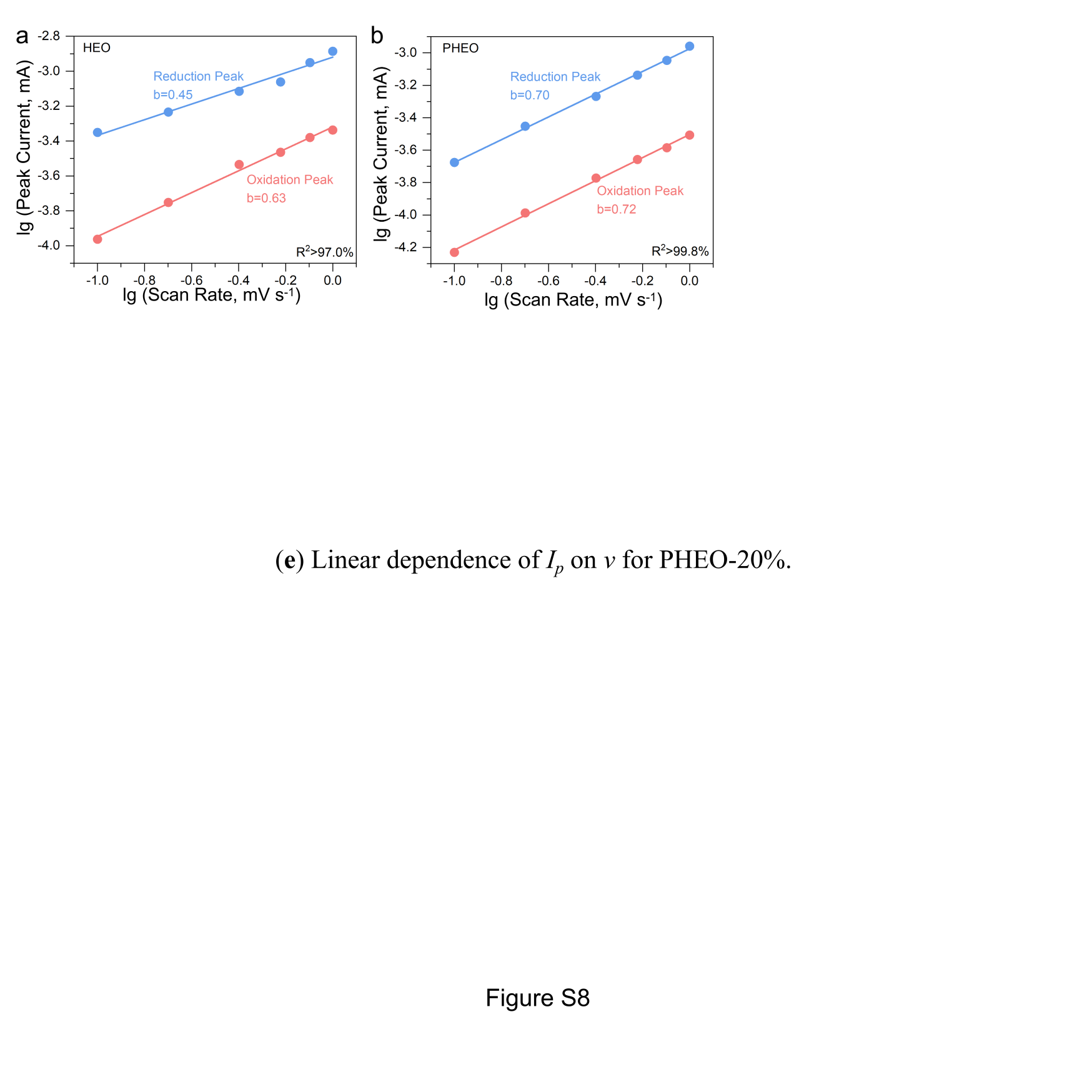


**Figure S9.** The linear relationship between peak current and scan rate of (a) HEO and (b) PHEO.


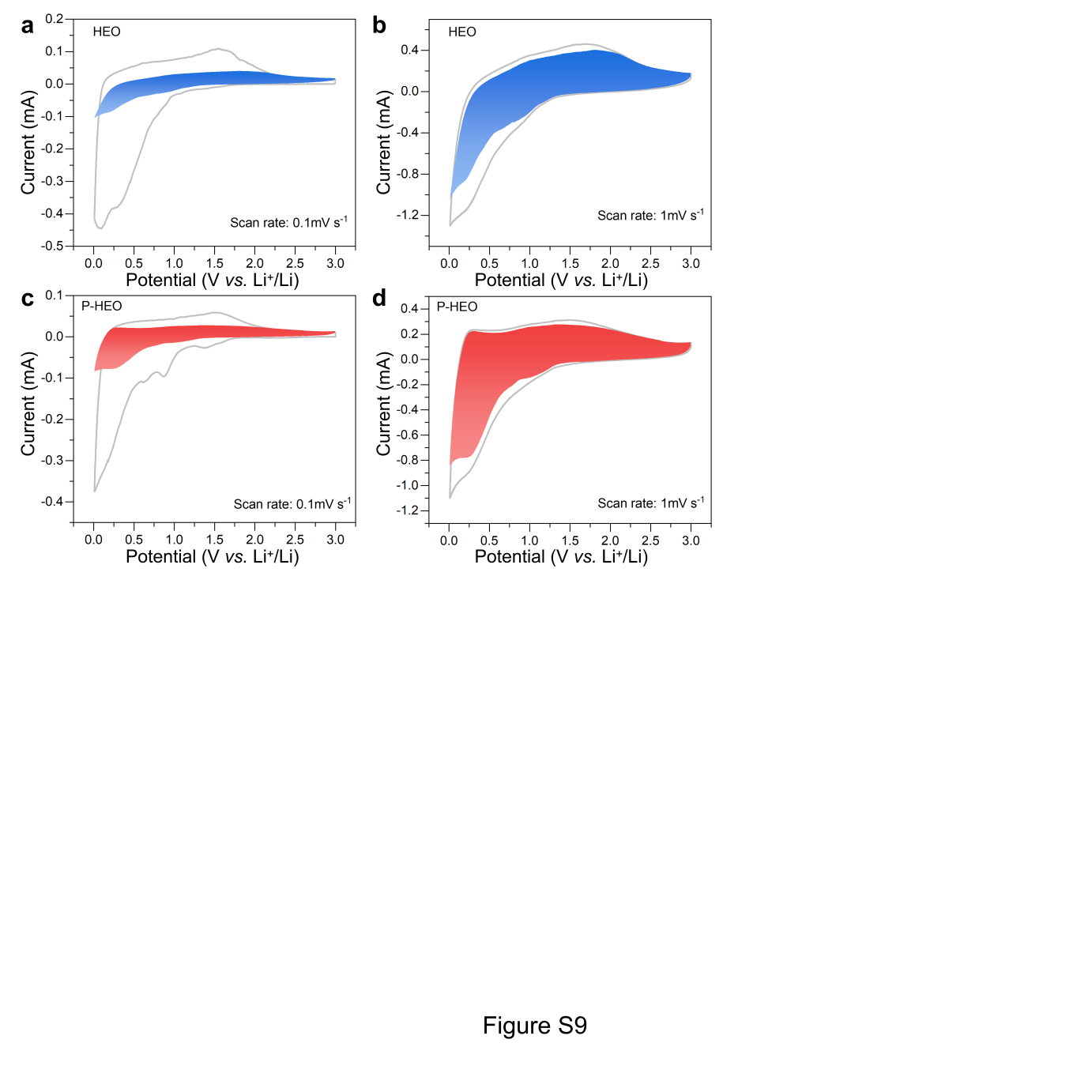


**Figure S10.** The capacitive contribution of HEO and P-HEO at (a,c) 0.1 mV s^-1^ and (b,d) 1 mV s^-1^.


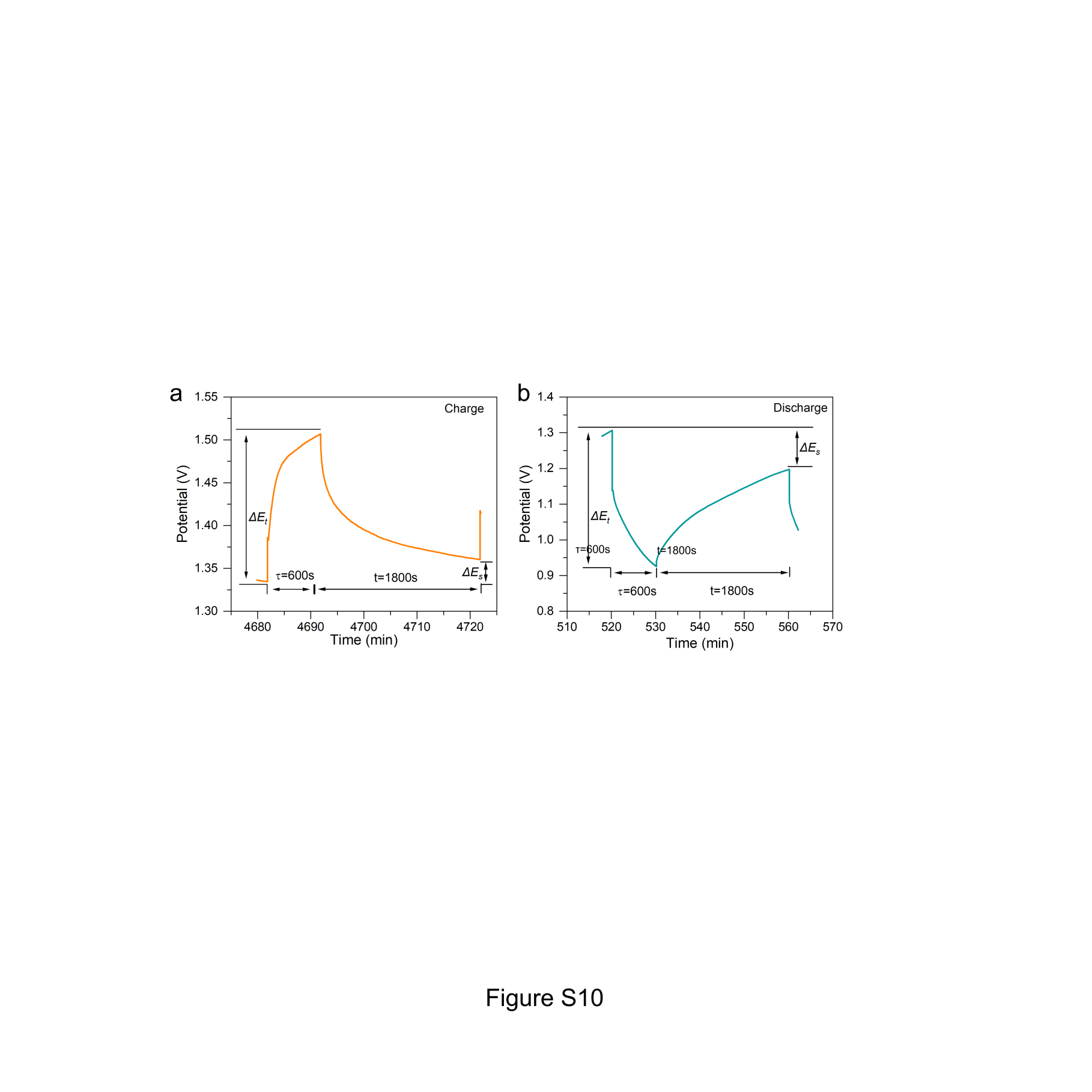


**Figure S11.** GITT curve of (a) charge and (b) discharge processes of PHEO.


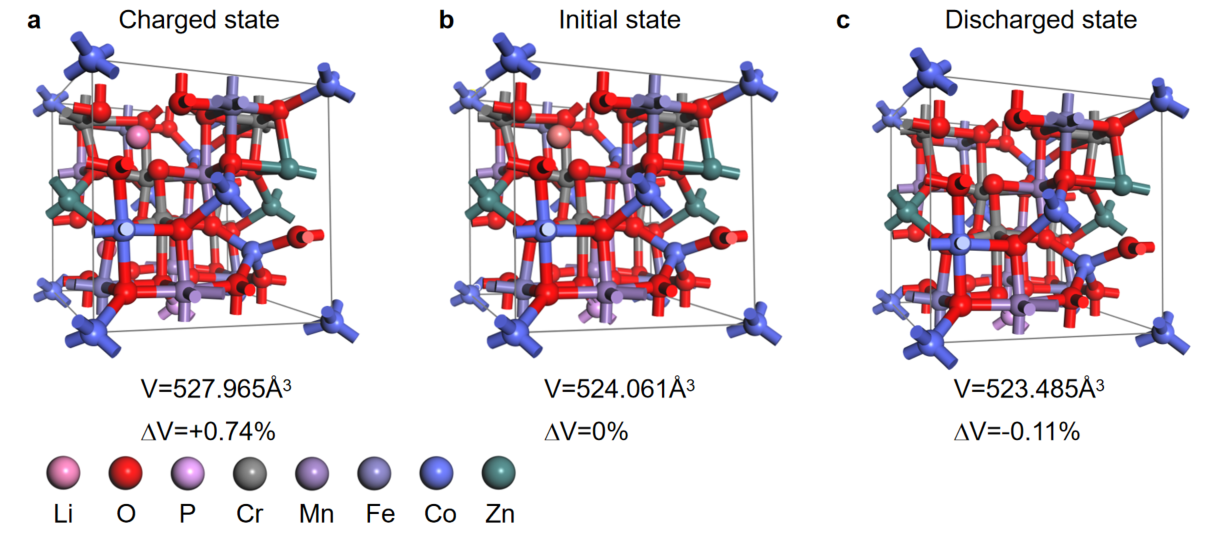


Figure S12. The volume of PHEO at (a) charged state, (b) initial state, and (c) discharged state.

Table S1. Molar ratio of precursors for HEO and PHEO preparation.

|  | **HEO** | **PHEO-10%** | **PHEO-20%** | **PHEO-30%** |
| --- | --- | --- | --- | --- |
| **LiNO_3_** | 1 mmol | 1 mmol | 1 mmol | 1 mmol |
| **Cr(NO_3_)_3_·9H_2_O** | 1 mmol | 1 mmol | 1 mmol | 1 mmol |
| **Mn(NO_3_)_2_·6H_2_O** | 1 mmol | 1 mmol | 1 mmol | 1 mmol |
| **Fe(NO_3_)_3_·9H_2_O** | 1 mmol | 1 mmol | 1 mmol | 1 mmol |
| **Co(NO_3_)_2_·6H_2_O** | 1 mmol | 1 mmol | 1 mmol | 1 mmol |
| **ZnCl_2_** | 1 mmol | 1 mmol | 1 mmol | 1 mmol |
| **NH_4_H_2_PO_4_** | 0 | 0.6 mmol | 1.2 mmol | 1.8 mmol |
| **C_6_H_8_O_7_** | 6 mmol | 6 mmol | 6 mmol | 6 mmol |

Table S2. Crystal structure parameters of PHEO.

|  | **a=b=c/Å** | **V/Å^3^** | **(311) FWHM/°** |
| --- | --- | --- | --- |
| **HEO** | 8.333255 | 578.69 | 0.258 |
| **P-HEO-10%** | 8.254311 | 562.40 | 0.296 |
| **P-HEO-20%** | 8.247279 | 560.96 | 0.440 |
| **P-HEO-30%** | 8.325278 | 577.03 | 0.260 |

Table S3. The percentage of different elements in PHEO and HEO.

| **Sample** | **Valence states and percentage/%** | | | | | | | | | | | |
| --- | --- | --- | --- | --- | --- | --- | --- | --- | --- | --- | --- | --- |
|  | **Cr** | | **Mn** | | **Fe** | | **Co** | | **Zn** | **O** | | |
|  | 3+ | 6+ | 3+ | 4+ | 2+ | 3+ | 2+ | 3+ | 2+ | O_L_ | O_V_ | O_A_ |
| **PHEO** | 85.68 | 14.28 | 30.83 | 69.17 | 59.19 | 40.81 | 26.68 | 73.32 | 100 | 12.46 | 64.01 | 23.54 |
| **HEO** | 15.32 | 84.68 | 37.66 | 62.34 | 55.20 | 44.80 | 52.12 | 47.88 | 100 | 56.49 | 41.77 | 1.74 |

Table S4. The Li^+^ diffusion coefficients of HEO and PHEO obtained from the CV curves.

| **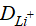/cm^2^ s^-1^** | **Reduction Peak (Intercalation)** | **Oxidation Peak (de-intercalation)** |
| --- | --- | --- |
| **HEO** | 4.82×10^-14^ | 4.38×10^-15^ |
| **PHEO** | 9.35×10^-14^ | 8.88×10^-15^ |
